# Highlight: Gene Cluster Reshuffling Drives Natural Sunscreen Evolution in Lichens

**DOI:** 10.1093/gbe/evad012

**Published:** 2023-02-08

**Authors:** Casey McGrath

**Affiliations:** Biology, Indiana University, Bloomington

*A new study reveals that the evolution of sunscreen pigments in lichen-forming fungi has been governed by the reshuffling of existing enzyme genes and novel accessory genes into new gene clusters.*

Lichens are diverse and colorful organisms that can be found in nearly every environment on Earth, from the arctic tundra to tropical rainforests. Due to the wide variety of their phenotypes and propensity to be misidentified as plants, fungi, or mosses, lichens have long been poorly understood. Lichens are composed of multiple distinct species, including at least one fungus and at least one photosynthetic partner, usually a green alga or cyanobacterium. According to Theo Llewellyn, a PhD candidate at Imperial College London and the Royal Botanic Gardens, Kew, “Lichens are hugely important for Earth's ecosystems and provide fantastic study systems for exploring many biological questions. However, they are understudied and often overlooked, meaning that much less is known compared to other organism groups, especially in the field of genomics.” Llewellyn is doing his part to change this by focusing his PhD work on lichen-forming fungi, which are known to produce a huge variety of bioactive secondary metabolites.

**Fig. 1.**
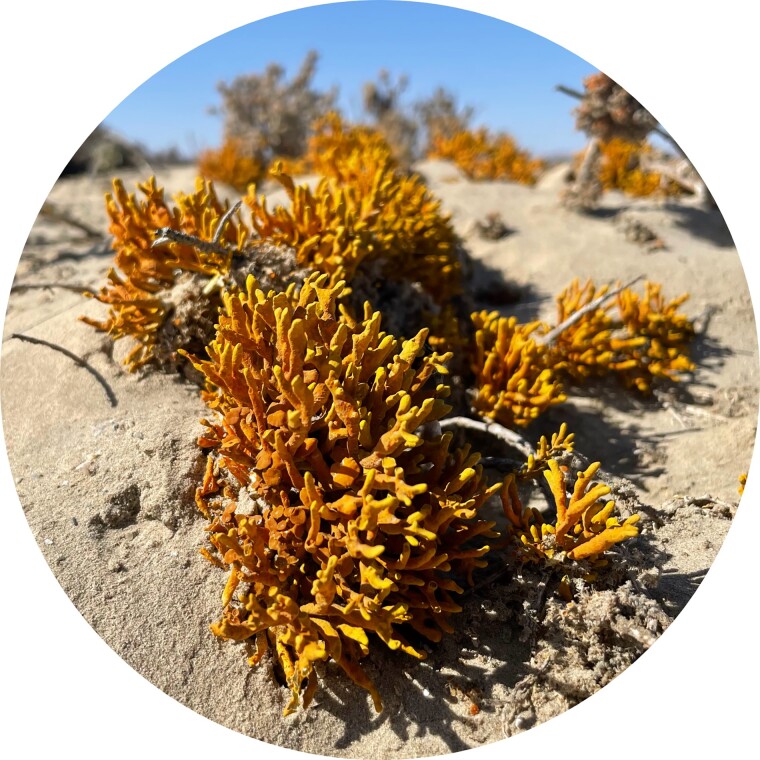
*Xanthodactylon flammeum* lichen (Teloschistaceae, Ascomycota) in Namib-Naukluft National Park, Namibia. Lichens of the Teloschistaceae family produce high concentrations of UV-protectant secondary metabolites, which allow them to survive in exposed, arid ecosystems across the globe. Photograph by Theo Llewellyn.

In a recent article published in *Genome Biology and Evolution*, Llewellyn and his colleagues in the United Kingdom, Brazil, and Israel investigated the evolution of orange “sunscreen” pigments known as anthraquinones in the *Teloschistales*, a diverse group of lichen-forming fungi (Llewellyn et al. 2023). During the Late Cretaceous, members of this lichen group switched from shaded forest habitats to exposed rocky ones. As shown in a 2015 paper by Ester Gaya—one of Llewellyn's PhD advisors and a senior author on the current paper—this habitat switch coincided with expanded production of UV-absorbing anthraquinones (Gaya et al. 2015), which allowed them to inhabit sunny and arid ecosystems worldwide (fig. 1). The new study in *GBE* demonstrates that there is a large diversity of anthraquinone biosynthesis genes among the *Teloschistales* and that their evolution has been governed largely by the reshuffling of existing enzyme genes and novel accessory genes into new gene clusters.

In fungi, the production of secondary metabolites is often driven by sets of genes that cluster together on the chromosome. These biosynthetic gene clusters (BGCs) represent hotspots of variation in the genome. Llewellyn and colleagues set out to conduct a comparative analysis to better understand the evolution of BGCs involved in anthraquinone biosynthesis among lichens. Unfortunately, there are relatively few published genomes of lichen-forming fungi due to the difficulty in growing the fungi in isolation. Therefore, Llewellyn et al. implemented a metagenomics approach to sequence and assemble 24 new lichen genomes, with a special emphasis on the *Teloschistales*, a large order of lichenized fungi. The authors then compared these genomes with 21 published genomes from other members of the *Lecanoromycetes*, the largest class of lichenized fungi.

The authors identified four BGC families involved in anthraquinone production across the sampled fungi. All four families were present in the *Teloschistales* clade, and all *Teloschistales* genomes contained at least one anthraquinone cluster. Interestingly, nearly all of the *Teloschistales* BGCs had a conserved four-gene organization that included the core anthraquinone synthase gene itself, as well as a thioesterase thought to cleave the final compound from the PKS enzyme, a dehydratase, and a unique ATP-binding cassette (ABC) transporter protein. Although homologs of each of the first three genes could be found in some species outside the *Teloschistales*, the ABC transporter was unique to this group, suggesting a history of genomic reshuffling in the *Teloschistales* to combine the novel accessory gene with existing anthraquinone enzyme genes.

Llewellyn and his colleagues believe the *Teloschistales* ABC transporter gene provides a critical clue to understanding the evolution of anthraquinones in these lichens. In fungi, transporters are often used to pump metabolites out of the cell before they can accumulate and become toxic. According to Llewellyn, “The discovery of an ABC transporter gene within the pigment gene cluster was a particular surprise. It had always puzzled us how these lichens were able to produce such large quantities of toxic orange pigments without poisoning themselves. Finding this unique transporter gene within the pigment gene cluster provided the first potential hypothesis for toxicity avoidance in this group.” The addition of this transporter to the anthraquinone BGC may explain how these lichens are able to accumulate such large amounts of anthraquinone crystals in their thallus and reproductive structures, which ultimately allowed them to expand into new environments.

The authors are now planning additional studies to glean even more insight into anthraquinone BGC evolution. “A related topic we are now exploring is whether these compounds may have additional functions beyond UV protection,” says Llewellyn. “For example, they are known to be cytotoxic to some fungi, and knowing how self-resistance is achieved within the *Teloschistales* may provide further insights into their biosynthetic evolution.” Llewellyn anticipates some challenges with this line of inquiry. “A major obstacle to this and lichen biology more generally is the difficulty of culturing lichenized fungi for experimental work. Their slow growth and resistance to being isolated from symbionts means that standard in vitro experimental designs tend not to work with lichens. Therefore, new approaches will need to be developed and tested before we can answer some of these questions.”

Additional taxonomic sampling and metagenomic analyses of more lichen genomes are also critical. “Given that the *Teloschistales* is such a diverse group,” notes Llewellyn, “our new genomic data only scratches the surface of their genomic and metabolic diversity. Our results are therefore a starting point that will need to be further explored when we are able to generate whole genome sequences for all major lineages within the clade.” As members of the *Teloschistales* can be found worldwide, sometimes in remote and hard-to-reach locations, a sustained effort among international partners and collaborators will be needed to ultimately achieve this goal.
